# Brotherhood

**Published:** 1911-08

**Authors:** G. Henri Bogart

**Affiliations:** Terre Haute, Ind.


For Texas Medical Journal.

### Brotherhood.

BY G. HENRI BOGART, TERRE HAUTE, IND.

Back, today, from the realm of dreams,
Filled with rare visioned joy, that streams
In glowing gleams
Of brotherhood:
Back to the day's prosaic toil,
Back to the strife
Of daily life—
War primeval as cave men waged—
Seeking a fellow's gain, as spoil,
Not with the club, or tooth, or claw;
By the more merciless clutch of law,
Plying a neighbor,
For fruits of labor—
Oh, I am sick of life's war as waged—
Wringing of hearts
In busy marts.
Wringing out gold, with bloodstained smears,
Washed from its heaps, with bitter tears;
Better the rage
Of other age;
When war was open, and dared not sneak,
Smiling, soft-spoken, traitorous, weak,
Into confiding hearts to grasp
Ultimate drops e'en to dying gasp.
Vampirish greed?
Money mad creed—
God, how we need

The living of "neighbor we love as self"
Rather than strife for that brother's pelf.
Would we could realize
All that glad enterprise,
"Loving one's neighbor as self."
Oh, I am glad of that realm of dreams,
Bright with its visioned hope, that streams,
In glowing gleams
Of brotherhood.

May 27, 1911.

